# Water/Ionic Liquid Hybrid Electrolyte for High Cycling Stability and Low‐Temperature Adaptability in Aqueous Lithium‐Ion Battery

**DOI:** 10.1002/advs.77610

**Published:** 2026-09-10

**Authors:** Jiwei He, Le Zhou, Mengmeng Cong, Keqiao Li, Donggyu Lee, Jiacheng Ji, Jinyu Deng, Zhigang Li, Baoling Huang

**Affiliations:** ^1^ Department of Mechanical and Aerospace Engineering The Hong Kong University of Science and Technology Kowloon Hong Kong China; ^2^ HKUST Shenzhen‐Hong Kong Collaborative Innovation Research Institute Futian Shenzhen China; ^3^ The Hong Kong University of Science and Technology Foshan Research Institute for Smart Manufacturing Kowloon Hong Kong China

**Keywords:** antifreeze additive, aqueous lithium‐ion batteries, imidazolium ionic liquids, interfacial regulation, low‐temperature electrolyte

## Abstract

Aqueous lithium‐ion batteries (ALIBs) often suffer from water crystallization and suppressed ionic transport under subzero conditions as well as narrow electrochemical stability windows. Here, we report a hybrid electrolyte based on 1 M LiNO_3_ in [EMIM][DEP]_0.1_‐(H_2_O)_0.9_ that combines the non‐flammability of ionic liquids with the high ionic conductivity of water. Strong [DEP]‐(H_2_O) hydrogen bonding disrupts the water network, lowering the freezing point from −24°C to −56°C while maintaining an ionic conductivity of 1.295 × 10^−3^ S cm^−1^ at −35°C. Meanwhile, ionic‐liquid enrichment at the anode forms a water‐restricted interphase that widens the apparent electrochemical stability window from 1.4 to 2.15 V, as supported by experimental characterization and molecular dynamic simulations. In a LiMn_2_O_4_/LiTi_2_(PO_4_)_3_ full cell, this hybrid electrolyte delivers 90 mAh g^−1^ at −35°C and 0.2 C and retains 90% of its initial capacity after 700 cycles at −35°C and 0.5 C, with an average Coulombic efficiency of 99.86%. Adding 0.05 M ZnSO_4_ further stabilizes the electrode/electrolyte interface, enabling 80% retention after 1400 cycles at 4 C. These results demonstrate a practical route to safe aqueous batteries with low‐temperature adaptability and long cycle life.

## Graphical abstract

A dilute hybrid electrolyte containing only 10 mol% [EMIM][DEP] simultaneously reorganizes the water hydrogen‐bond network and creates an ionic‐liquid‐rich region at the anode. This coupled bulk and interfacial regulation suppresses freezing and hydrogen evolution, broadens the electrochemical stability window, and enables an aqueous LMO/LTP full cell to retain 90% capacity after 700 cycles at −35°C.

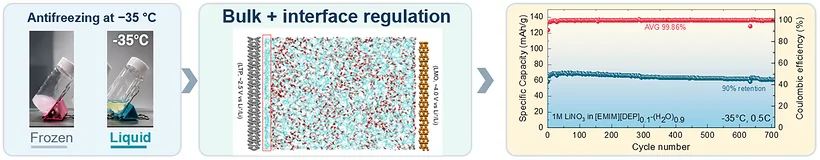

## 1 Introduction

In recent years, aqueous lithium‐ion batteries (ALIBs) have gained increasing attention as promising alternatives to conventional organic electrolyte‐based lithium‐ion batteries for large‐scale energy storage due to rapidly growing safety concerns [1, 2, 3, 4]. Unlike organic electrolytes, which pose risks of flammability and instability, aqueous electrolytes provide a non‐flammable and high ion‐conductivity solution for energy storage applications [5, 6]. Their inherent high safety, low cost, and environmental friendliness make ALIBs ideal for portable or wearable electronics as well as large‐scale energy storage systems. However, aqueous lithium‐ion batteries are facing several serious challenges, particularly short lifespan and poor low‐temperature performance, due to the narrow electrochemical stability window (ESW) and the high freezing point of the traditional aqueous electrolytes [5, 6, 7], which greatly limit the practical applications of ALIBs in cold‐climate regions. At subzero temperatures, conventional aqueous electrolytes suffer from water crystallization and increased electrolyte resistance, leading to severe capacity fading, sluggish ion transport, and poor cycle stability [7, 8, 9]. Therefore, developing a low‐temperature‐resistant aqueous electrolyte is crucial for expanding the usability of ALIBs under extreme conditions.

Ice nucleation, as the initial stage of freezing, occurs when water molecules with tetrahedral coordination organize into stacked hexagonal sequences, ultimately forming an ordered ice structure [10, 11, 12]. Once ice forms, the number of free water molecules capable of solvating and transporting ions is significantly reduced. Therefore, to maintain ion transport and battery performance in cold environments, it is essential to prevent water from forming a crystalline structure [10, 11, 12]. One effective strategy is the use of high‐concentration “water‐in‐salt” electrolytes [13, 14], such as 21 M lithium bis(trifluoromethanesulfonyl)imide (LiTFSI), resulting in a reversible capacity of 111 mAh g^−1^ at 0.2C at −20°C [15]. However, at ultralow temperatures, the solubility of the salt decreases significantly, causing salting‐out effects that severely limit its practical application. Another approach is to mix organic co‐solvents with water, such as dimethyl sulfoxide (DMSO), yielding a capacity at −50 °C that is 60% of the value obtained at 25 °C [7]. However, to achieve a low freezing point, the concentrations of these organic co‐solvents are generally high (over 30% for DMSO [7]), making the electrolyte flammable and environmentally hazardous. Therefore, a balanced approach is needed to ensure safety, maintain good ionic conductivity, broaden the ESW, and prevent freezing under low‐temperature conditions.

Ionic liquids (ILs) are now widely used as co‐solvents in aqueous Zn‐ion batteries to improve the room‐temperature performance due to their low volatility, high thermal and electrochemical stability, and non‐flammability [16]. The ionic liquid 1‐hydroxyethyl‐3‐methylimidazolium triflate (HO‐EMImTfO) has been used as an additive to form a protective layer on Zn, enabling cells with 99.5% Coulombic efficiency for 1000 cycles [17]. The ionic liquid 1‐ethyl‐3‐methylimidazolium bis(fluorosulfonyl)amide ([Emim][FSI]) forms an “ionic‐liquid‐water pocket” electrolyte with H_2_O/Zn(OTf)_2_, leading to 87.7% capacity retention after 500 cycles [18]. Recent studies have further shown that ionic liquid‐containing aqueous electrolytes can sustain electrochemical operation across wide temperature ranges by simultaneously regulating water structure and electrode interfaces [18, 19, 20, 21]. These results reinforce the potential of ionic‐liquid/water hybridization as a general strategy for wide‐temperature aqueous energy‐storage systems.

Herein, we developed a hybrid aqueous/ionic liquid electrolyte based on 1 M LiNO_3_ in [EMIM][DEP]_0.1_‐(H_2_O)_0.9_ to address the coupled challenges of freezing, narrow ESW, and limited cycle life in ALIBs. In this system, the DEP^−^ anion forms strong hydrogen bonds with water and breaks the original water–water network, lowering the freezing point of the electrolyte to about −56°C while maintaining sufficient ionic conductivity at −35°C. At the same time, EMIM^+^ cations and DEP^−^ anions accumulate at the anode to form an ionic‐liquid‐rich interphase that repels water from the electrode surface and extends the ESW from ∼1.4 V for 1 M LiNO_3_ in water to ∼2.15 V for the hybrid electrolyte. Both spectroscopic characterization (FTIR, ^1^H NMR) and molecular dynamics simulations are used to clarify the bulk hydrogen‐bond network and interfacial structure responsible for these effects. When applied in an LMO/LTP full cell, the hybrid electrolyte enables high Coulombic efficiency and excellent cycling stability at −35°C, and, with a small ZnSO_4_ additive, delivers long cycle life and high rate capability at room temperature. This work establishes a general strategy for designing IL/H_2_O hybrid electrolytes that simultaneously achieve a wide ESW, high low‐temperature Coulombic efficiency, and durable operation.

## 2 Results and Discussion

**FIGURE 1:**
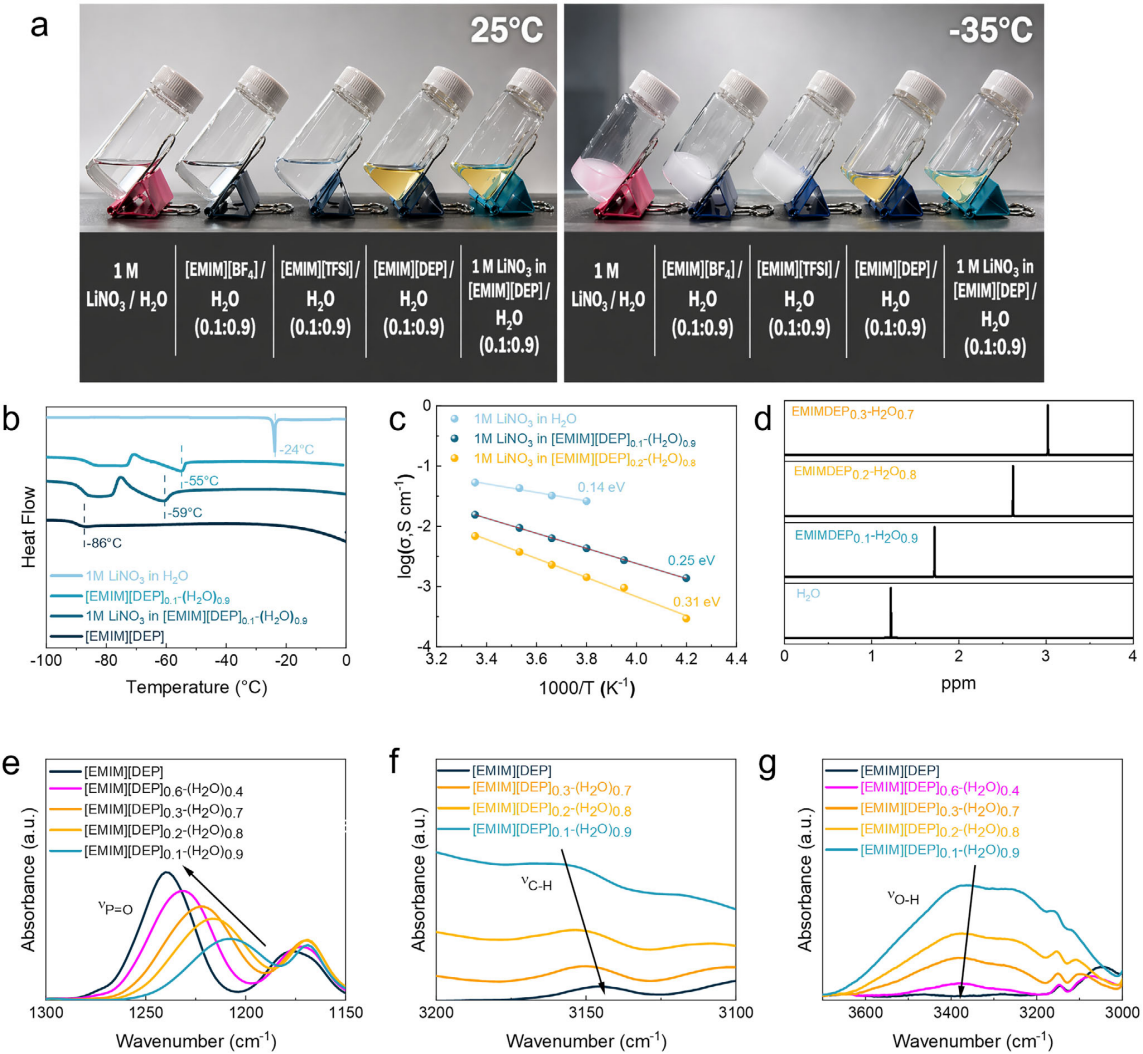
The properties of the hybrid electrolyte. (a) Photographs of different ionic liquid/water mixtures after 1 h at −35 °C, demonstrating their physical states. From left to right: 1 M LiNO_3_ in water, [EMIM][BF_4_]_0.1_‐(H_2_O)_0.9_, [EMIM][TFSI]_0.1_‐(H_2_O)_0.9_, [EMIM][DEP]_0.1_‐(H_2_O)_0.9_ and 1 M LiNO_3_ in [EMIM][DEP]_0.1_‐(H_2_O)_0.9_. (b) DSC curves of [EMIM][DEP]‐H_2_O mixtures with different molar ratios and 1 M LiNO_3_ in water, showing their respective freezing points. (c) Temperature‐dependent ionic conductivity of [EMIM][DEP]‐H_2_O mixtures with different molar ratios and 1 M LiNO_3_ in water. (d) The ^1^H NMR chemical shifts of different mole fractions [EMIM][DEP] of aqueous [EMIM][DEP] solutions. (e–g) FTIR spectra of [EMIM][DEP]‐H_2_O mixtures with varying [EMIM][DEP] molar fractions: (e) P═O asymmetric stretching band; (f) C_2_‐H stretching band in the EMIM^+^ cation; (g) O‐H stretching band of water.

The freezing points of the water/ionic liquid mixtures usually depend more on the anions of the ionic liquid [25, 26]. To identify a suitable ionic liquid for low‐temperature aqueous lithium‐ion batteries, several EMIM‐based ionic liquids with different anions ([EMIM][BF_4_], [EMIM][TFSI] and [EMIM][DEP]) were each mixed with water at a 1:9 molar ratio, and their physical states were monitored after storage at 0, −10, −25 and −35°C for 1 h (Figure 1a and Figure S1). All candidates remained liquid down to −20°C. At −25°C, 1 M LiNO_3_ in pure water and the [EMIM][TFSI]‐H_2_O mixture were already frozen, and at −35°C the [EMIM][BF_4_]‐H_2_O mixture also solidified, whereas the [EMIM][DEP]_0.1_‐(H_2_O)_0.9_ mixture remained fully liquid. These observations indicate that [EMIM][DEP]_0.1_‐(H_2_O)_0.9_ possesses superior anti‐freezing characteristics and is a promising additive for low‐temperature electrolytes. Importantly, dissolving 1 M LiNO_3_ in [EMIM][DEP]_0.1_‐(H_2_O)_0.9_ did not cause salting‐out or phase separation, confirming good salt solubility and phase stability under subzero conditions. To evaluate whether the operating limit could be extended by changing the lithium salt, Li_2_SO_4_, LiTFSI, and LiCl were screened in the same hybrid solvent. Li_2_SO_4_ could not be completely dissolved at 1 M at room temperature. (Figure S2) LiTFSI dissolved at room temperature but visibly precipitated after storage at −35°C, (Figure S3a), indicating insufficient low‐temperature solubility. In contrast, 1 M LiCl in [EMIM][DEP]_0.1_‐(H_2_O)_0.9_ exhibited a freezing transition at approximately −86°C and no visible precipitation over the investigated temperature range (Figure S3b,c). However, Cl^−^ can disrupt passive films and promote pitting corrosion of stainless steel [22], making current‐collector compatibility a practical limitation in the present cell configuration. Therefore, LiNO_3_ was retained because it provides a more favorable balance of solubility, low‐temperature phase stability, and compatibility with the cell components. Therefore, LiNO_3_ was chosen as the lithium salt by considering both solubility and economic feasibility. On the other hand, increasing the [EMIM][DEP] content will reduce the solubility of LiNO_3,_ and 1 M LiNO_3_ can hardly dissolve when the [EMIM][DEP] content is above 20 mol%. The freezing behaviors of pure [EMIM][DEP], [EMIM][DEP]_0.2_–(H_2_O)_0.8_ and [EMIM][DEP]_0.1_‐(H_2_O)_0.9_ mixtures and the corresponding electrolytes containing 1 M LiNO_3_ were further quantified by differential scanning calorimetry (DSC, Figure 1b and Figure S4). Pure [EMIM][DEP] showed a freezing point of −86°C, while [EMIM][DEP]_0.2_‐(H_2_O)_0.8_ and [EMIM][DEP]_0.1_‐(H_2_O)_0.9_ exhibited freezing points of −73°C and −56°C, respectively. For comparison, 1 M LiNO_3_ in water froze at −24°C, whereas 1 M LiNO_3_ in [EMIM][DEP]_0.1_‐(H_2_O)_0.9_ and 1 M LiNO_3_ in [EMIM][DEP]_0.2_‐(H_2_O)_0.8_ showed freezing points of −59°C and −76°C, respectively. These results confirm that increasing the [EMIM][DEP] content effectively depresses the freezing point of both the solvent and the LiNO_3_‐based electrolyte. However, low‐temperature battery performance is not only determined by the freezing point but also strongly influenced by ionic conductivity. Therefore, the temperature‐dependent ionic conductivities of 1 M LiNO_3_ in water, 1 M LiNO_3_ in [EMIM][DEP]_0.1_‐(H_2_O)_0.9_, and 1 M LiNO_3_ in [EMIM][DEP]_0.2_‐(H_2_O)_0.8_ were measured (Figure 1c and Figure S5). As expected, the higher viscosity of [EMIM][DEP] leads to lower conductivity in the hybrid electrolytes than in the pure aqueous LiNO_3_ solution at room temperature [23, 24]. With decreasing temperature, all electrolytes show reduced ionic conductivity due to the lower mobility of charge carriers. Notably, 1 M LiNO_3_ in [EMIM][DEP]_0.1_‐(H_2_O)_0.9_ maintains an ionic conductivity of 1.295 ×  10^−3^ S cm^−1^ at −35°C, whereas the conductivity of the [EMIM][DEP]_0.2_‐(H_2_O)_0.8_‐based electrolyte falls below 1 × 10^−3^ S cm^−1^ at temperatures below −20°C. Arrhenius fitting further shows that the activation energy for ionic conduction is 0.25 eV for 1 M LiNO_3_ in [EMIM][DEP]_0.1_‐(H_2_O)_0.9_, which is lower than that of 1 M LiNO_3_ in [EMIM][DEP]_0.2_‐(H_2_O)_0.8_ (0.31 eV), indicating a lower energy barrier for ion transport in the optimized composition. This comparison shows that [EMIM][DEP]_0.1_‐H_2_O_0.9_ provides a better balance between freezing‐point depression and ionic conductivity and was therefore selected as the optimized hybrid solvent for subsequent studies.

To clarify how [EMIM][DEP] lowers the freezing point and changes the properties of water, the molecular interactions in the hybrid aqueous/[EMIM][DEP] electrolyte were investigated by Fourier‐transform infrared (FTIR) spectroscopy (Figure 1e–g). The FTIR spectra focus on three key vibrations that are sensitive to hydrogen bonding: the P═O stretching mode of the DEP^−^ anion, the C2‐H stretching mode of the EMIM^+^ cation, and the O─H stretching band of water. A more detailed FTIR analysis is provided in Figure S6. As shown in Figure 1e, the band at 1207 cm^−1^ corresponds to the asymmetric P═O stretching vibration in DEP^−^ [25, 26]. With increasing [EMIM][DEP] content, this band shifts to a higher wavenumber, from 1207 to 1240 cm^−1^, indicating stronger hydrogen bonding between the oxygen atom in P═O and the hydrogen in water (P═O···H─O). This pronounced blue shift shows that hydrogen bonding between water and the DEP^−^ anion is a dominant interaction in the hybrid electrolyte. Water molecules also interact with the EMIM^+^ cation. The band at 3155 cm^−1^, assigned to the C2‐H stretching vibration in EMIM^+^ [27], shifts slightly to a lower wavenumber (from 3155 to 3144 cm^−1^) as the [EMIM][DEP] content increases from 10 to 100 mol%. This red shift reflects the formation of C2‐H···O─H hydrogen bonds between EMIM^+^ and water. However, the change in the C2‐H band is much smaller than that of the P═O band, indicating that water molecules form stronger and more extensive hydrogen bonds with the anions than with the cations. The O─H stretching region in Figure 1g further supports this conclusion. As more [EMIM][DEP] is added, the O─H band moves to a higher wavenumber, which means that hydrogen bonding among water molecules becomes weaker. In other words, water‐water hydrogen bonds are gradually replaced by water‐DEP^−^ and water‐EMIM^+^ bonds. This disruption of the original water network makes it more difficult for water molecules to arrange into an ordered ice structure and helps the electrolyte remain liquid at low temperatures.

To complement the FTIR results and further probe hydrogen‐bonding between water and the ionic liquid, ^1^H nuclear magnetic resonance (NMR) spectra were recorded for [EMIM][DEP]–H_2_O mixtures with different compositions (Figure 1d and Figures S7–S9). The ^1^H chemical shift of water is sensitive to its local hydrogen‐bond environment. In [EMIM][DEP]_0.3_‐(H_2_O)_0.7_, the water signal appears at 3.039 ppm and shifts progressively upfield to 2.657, 1.721, and 1.2 ppm for [EMIM][DEP]_0.2_‐(H_2_O)_0.8_, [EMIM][DEP]_0.1_‐(H_2_O)_0.9_, and pure H_2_O, respectively. This trend indicates that at higher [EMIM][DEP] contents (water‐deficient conditions), water molecules experience stronger interactions with the DEP^−^ anions, which act as hydrogen‐bond acceptors through their phosphate oxygen atoms, leading to proton deshielding and broader signals due to slower exchange dynamics [28]. As the water content increases, more water‐water hydrogen bonds form and the average interaction between water and DEP^−^ weakens, resulting in lower chemical shifts. These observations confirm the formation of strong, localized water‐anion interactions that dominate the chemical environment in [EMIM][DEP]‐rich mixtures and support the conclusion that [EMIM][DEP] significantly rewires the hydrogen‐bond network of water.

**FIGURE 2:**
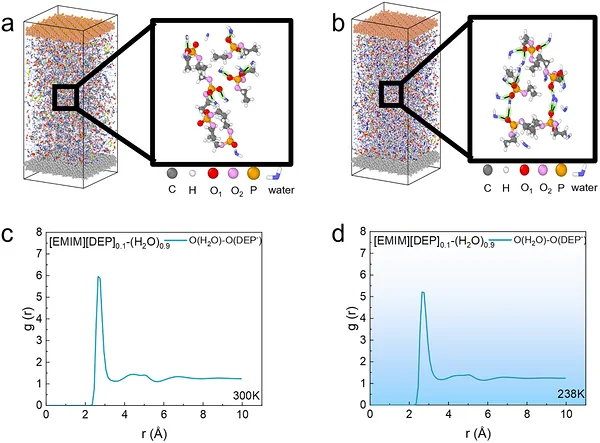
Study of the hydrogen bond in the hybrid electrolyte. MD simulation snapshots of [EMIM][DEP]_0.1_‐(H_2_O)_0.9_ at (a) 300K and (b) 238K. The RDFs of the oxygen atom in water molecules around the oxygen atom in [EMIM][DEP] at (c) 300K and (d) 238K.

To provide further insight into how these hydrogen‐bond interactions influence the freezing behavior of the solvent, molecular dynamics (MD) simulations were carried out for both pure water and [EMIM][DEP]_0.1_‐(H_2_O)_0.9_ at different temperatures (Figure 2 and Figure S10). For pure water, the molecules are randomly distributed in the simulation box at 300 K, consistent with a disordered liquid structure. In contrast, when the temperature is lowered to 238 K, water molecules arrange into a hexagonal crystalline structure, indicating ice formation. This structural transition is also reflected in the radial distribution functions (RDFs), where pure water exhibits a series of continuous sharp peaks at 238 K, characteristic of long‐range ordering in the ice phase. By comparison, in [EMIM][DEP]_0.1_‐(H_2_O)_0.9_, hydrogen bonds form between the hydrogen atoms of water and the oxygen atoms of the DEP^−^ anions at 300 K (Figure 2a), disrupting the original water‐water hydrogen‐bond network and weakening the tendency of water molecules to organize into an extended ice‐like structure. When the system is cooled to 238 K (Figure 2b), the hydrogen‐bond network between DEP^−^ and water remains intact, indicating that these interactions are robust and persist under subzero conditions. The RDFs provide further structural insight (Figure 2c,d). For [EMIM][DEP]_0.1_‐(H_2_O)_0.9_, the O(H_2_O)‐O(DEP^−^) RDF exhibits a pronounced coordination peak at ∼2.65 Å at both 300 and 238 K, with nearly unchanged peak position and intensity. The constant coordination distance confirms that the local chemical environment around water molecules remains stable upon cooling and that O(H_2_O)‐O(DEP^−^) interactions persist at low temperature. Together, the comparison with pure water shows that, whereas water alone readily forms an ordered hexagonal ice structure at 238 K, the presence of [EMIM][DEP] effectively rewires the hydrogen‐bond network of water and suppresses ice nucleation. This explains the pronounced freezing‐point depression of the [EMIM][DEP]‐based hybrid electrolyte and its importance for low‐temperature battery operation.

**FIGURE 3:**
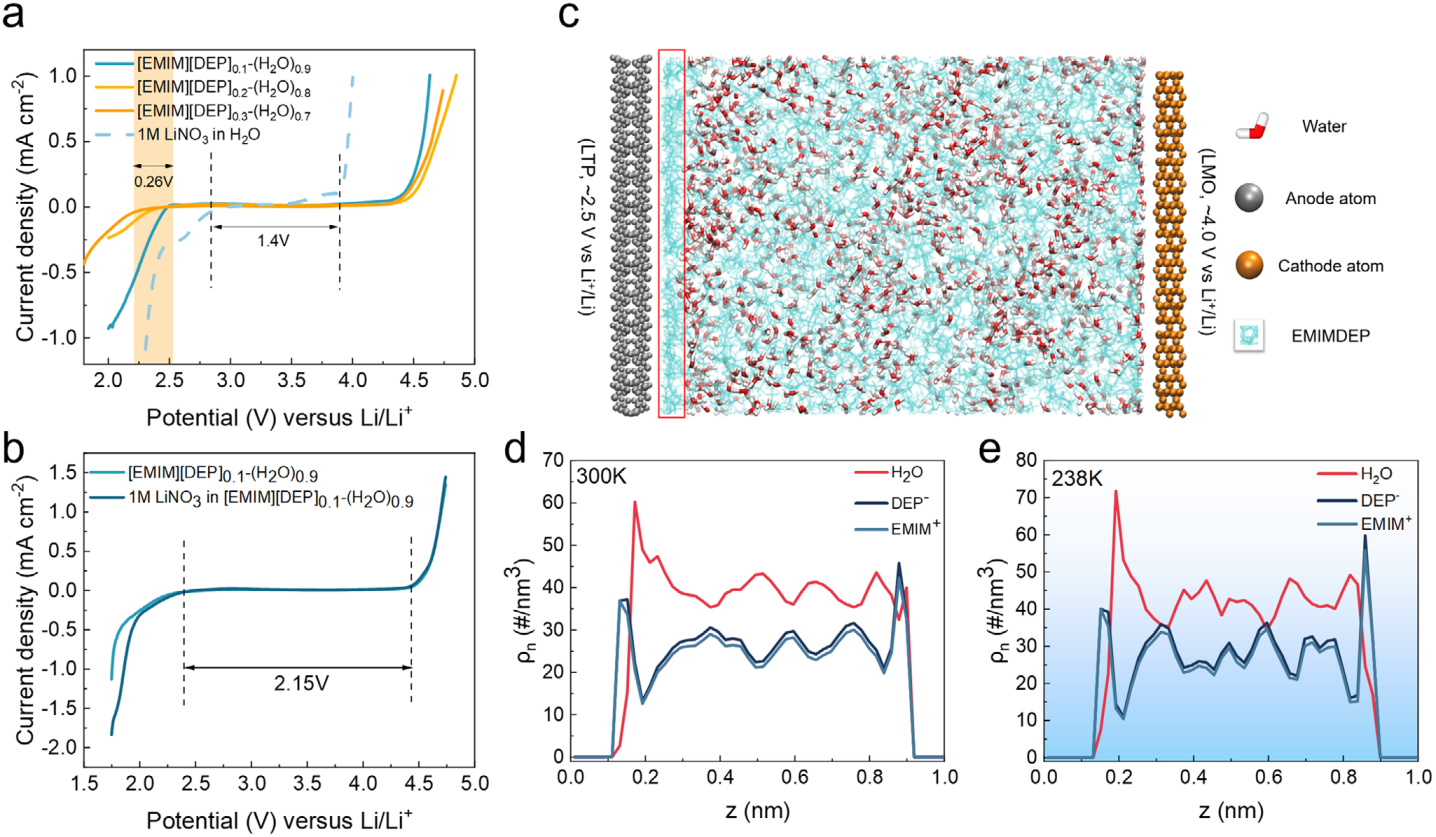
Electrochemical stability window and the MD simulation of the hybrid electrolyte. (a) ESW for [EMIM][DEP]‐H_2_O in different molar ratios. (b) ESW for [EMIM][DEP]‐H_2_O with different salt concentrations. (c) MD simulation snapshots of [EMIM][DEP]_0.1_‐(H_2_O)_0.9_ at 300K. Water and [EMIM][DEP] number density profiles at (d) 300K and (e) 238K.

The electrochemical stability window of the [EMIM][DEP]‐H_2_O‐based electrolytes was first evaluated by linear sweep voltammetry (LSV). LSV measurements were performed using stainless‐steel mesh as both working and counter electrodes and Ag/AgCl (3 M KCl) as the reference electrode at a scan rate of 1 mV s^−1^. For the conventional 1 M LiNO_3_ aqueous electrolyte, the electrochemical stability window is only about 1.4 V (Figure S11). In contrast, introducing [EMIM][DEP] into the electrolyte clearly widens the window, indicating that the hybrid electrolyte is more resistant to water decomposition (Figure 3a). When the [EMIM][DEP] content is increased from 10 to 30 mol%, the apparent electrochemical window further expands to about 2.15 V. This enlargement mainly arises from a shift of the cathodic limit: the onset potential of the hydrogen evolution reaction (HER) moves from 2.25 V vs Li^+^/Li in [EMIM][DEP]_0.3_‐(H_2_O)_0.7_ to 2.51 V vs Li^+^/Li in [EMIM][DEP]_0.1_‐(H_2_O)_0.9_, while the anodic limit remains almost unchanged. These results indicate that increasing the ionic liquid fraction primarily suppresses HER on the negative side, rather than altering the oxidation stability on the positive side. The effect of LiNO_3_ on the electrochemical stability window was examined (Figure 3b). The addition of 1 M LiNO_3_ to [EMIM][DEP]_0.1_‐(H_2_O)_0.9_ only slightly changes the anodic and cathodic limits, indicating that LiNO_3_ has a negligible influence on the ESW compared with EMIMDEP.

To understand the origin of this HER suppression, molecular dynamics (MD) simulations were used to probe the interfacial structure of the electrolyte in the LMO/LTP full cell (Figure 3c and Figure S12). Here, the purpose of the MD simulation is not to directly reproduce the stainless‐steel surface used in the LSV measurement, but to provide insight into the electrode/electrolyte interfacial structure in the practical battery system. In the full cell, LTP operates at a lower potential of about 2.5 V vs Li^+^/Li, whereas LMO operates at a higher potential of about 4.0–4.1 V vs Li^+^/Li, and these different operating potentials lead to different interfacial ion organizations. As shown by the snapshots and number‐density profiles (Figure 3d), both EMIM^+^ cations and DEP^−^ anions accumulate in the interfacial region near the LTP anode, forming a compact ionic‐liquid‐rich layer, whereas no obvious accumulation of water is observed at the LMO side in the present system. Such preferential ionic‐liquid enrichment at the low‐potential LTP side can be understood from the potential‐dependent organization of the electrical double layer: once the electrode is polarized, the interfacial ionic structure responds strongly to the local electric field, and ions are arranged in a layered fashion near the surface rather than remaining homogeneously distributed in the bulk. Previous studies on water electrosorption in ionic liquids have shown that the interfacial distributions of ions and water are governed by the combined effects of electrode polarization, local electric field, water‐ion association, and available free space within the double layer, and that the ion layering itself is a direct response to electrification. In the present system, the lower‐potential LTP surface favors the accumulation of ionic‐liquid species in the near‐surface region; EMIM^+^ is electrostatically attracted toward the anode interface, while the resulting dense interfacial packing and ion–ion correlations allow DEP^−^ to remain in the same near‐surface region as part of a compact ionic‐liquid‐rich interphase, rather than forming a simple single‐ion layer. At the same time, our FTIR and NMR results show that water interacts more strongly with DEP^−^ than with EMIM^+^, so water near the anode is more likely to reside in ion‐coordinated environments than to directly contact the electrode surface. As a result, this compact interfacial structure effectively restricts the direct access of water molecules to the LTP surface and reduces the local activity of free water near the anode, thereby delaying the onset of HER. The same interfacial motif is preserved at lower temperatures, with slightly shorter intermolecular distances but the ionic liquid still dominating the anode‐side interfacial region, indicating that this stabilizing configuration is robust under subzero conditions (Figure 3e). Finally, the effect of LiNO_3_ on the electrochemical stability window was examined (Figure 3b). The addition of 1 M LiNO_3_ to [EMIM][DEP]_0.1_‐(H_2_O)_0.9_ only slightly changes the anodic and cathodic limits, indicating that LiNO_3_ has a negligible influence on the ESW compared with [EMIM][DEP].

**FIGURE 4:**
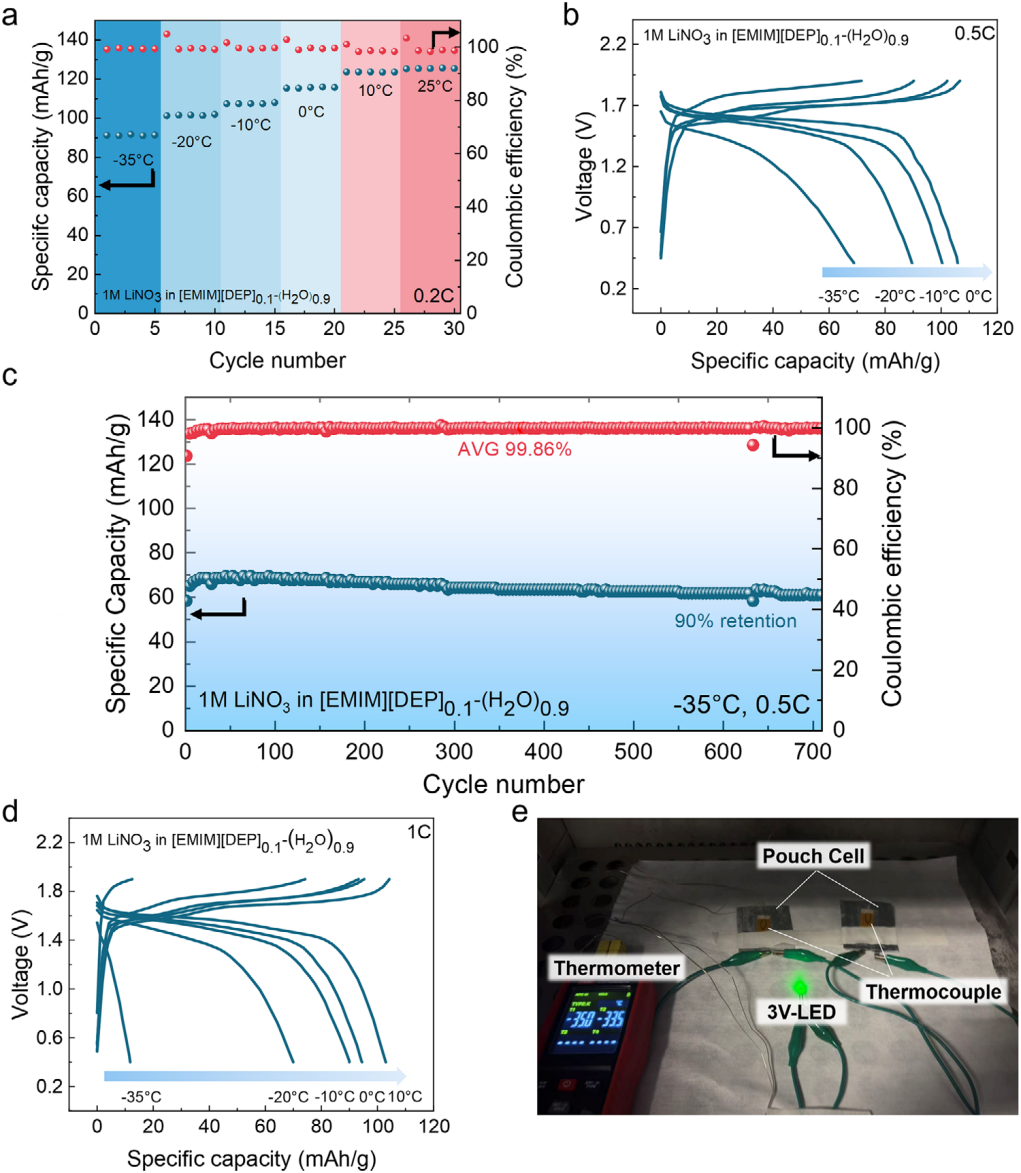
Electrochemical performance and application of full cells at low temperatures. (a) Electrochemical performance of full batteries with the hybrid electrolyte at 0.2C under different temperatures. (b) Cycling stability of the batteries using 1 M LiNO_3_ in [EMIM][DEP]_0.1_‐(H_2_O)_0.9_ at 0.5 C at 238K (c) Cycling performance of the full cell at 0.5C at 238K. (d) Galvanostatic charge–discharge profiles at different temperatures at 1C. (e) Demonstration of the battery providing power to a 3 V LED light.

With the expanded electrochemical stability window provided by the hybrid ionic liquid/aqueous electrolyte (2.35–4.42 V vs Li/Li^+^, Figure 3a), a full aqueous lithium‐ion battery was assembled using LiMn_2_O_4_ (LMO, ∼4.0 V vs Li^+^/Li) as the cathode and LiTi_2_(PO_4_)_3_ (LTP, ∼2.5 V vs Li^+^/Li, coated with 3 wt.% carbon) as the anode. The electrolyte was 1 M LiNO_3_ in [EMIM][DEP]_0.1_‐(H_2_O)_0.9_. The reversible electrochemical behavior of both electrodes in this hybrid electrolyte was confirmed by cyclic voltammetry (CV), as shown in Figure S13, where well‐defined and nearly overlapping redox peaks indicate good Li^+^ insertion/extraction reversibility at room temperature. To evaluate the low‐temperature performance of the aqueous lithium‐ion batteries (ALIBs), LMO/LTP full cells were tested in an environmental chamber over a temperature range from −35°C to 10°C. (Figure S14) Galvanostatic charge–discharge tests at 0.2 C (Figure 4a) show that the cells deliver discharge capacities of 90, 100, 105, 113, and 120 mAh g^−1^ at −35, −20, −10, 0, and 10°C, respectively. The gradual increase in capacity with temperature is consistent with the strong temperature dependence of the ionic conductivity of the electrolyte. At lower temperatures, the decrease in ionic conductivity and the increase in internal resistance limit Li^+^ transport, leading to reduced discharge capacity [29]. To further clarify this temperature‐dependent behavior, electrochemical impedance spectroscopy (EIS) was carried out at different temperatures (Figures S14 and S15). The temperature‐dependent separator‐only and full cell EIS spectra were fitted using the equivalent circuits shown in Figure S15. For 1 M LiNO_3_ in [EMIM][DEP]_0.1_‐H_2_O_0.9_, Rb increases from 2.74 at 25°C to 42.19 Ω at −35°C, while Rct, cell increases from 6.29 to 39.44 Ω. For the electrolyte containing 0.05 M ZnSO_4_, Rb increases from 2.84 Ω at 25°C to 34.39 Ω at −35°C, and Rct, cell increases from 8.91 to 45.35 Ω. The fitted resistance components are summarized in Table S2. Although [EMIM][DEP]_0.2_‐(H_2_O)_0.8_ has a lower freezing point than [EMIM][DEP]_0.1_‐(H_2_O)_0.9_, its lower ionic conductivity at low temperature becomes a limiting factor for battery performance (Figure S16). This is confirmed by testing an LMO/LTP full cell with 1 M LiNO_3_ in [EMIM][DEP]_0.2_‐(H_2_O)_0.8_ at −35°C and 0.2 C: the discharge capacity is only 40 mAh g^−1^, much lower than that obtained with [EMIM][DEP]_0.1_‐(H_2_O)_0.9_. These results demonstrate that, while freezing‐point depression is important, maintaining sufficient ionic conductivity is equally critical for practical low‐temperature operation. The rate performance of the optimized electrolyte was further examined at 0.5 C and 1 C (Figure 4b,d). At 0.5 C, the discharge capacity is 50 mAh g^−1^ at −35°C and increases to 90, 100, and 113 mAh g^−1^ at −20, −10, and 0°C, respectively. The discharge voltage plateaus remain well defined and do not show obvious deterioration at −20, −10, and 0°C, indicating that the hybrid electrolyte can still support relatively stable electrode reactions at these temperatures. We also test the battery low‐temperature performance of the electrolyte without the ionic liquid additive. Figure S17 shows that the discharge capacity at 0.5 C is 106 mAh g^−1^ at 25°C and decreases to 90, 60, and 34 mAh g^−1^ at 0, −10, and −20°C, respectively. Moreover, the discharge voltage plateau is lower than the 25°C. At 1 C, the capacity drops to 18 mAh g^−1^ at −35°C due to more severe kinetic limitations, but recovers to 70, 90, 97, and 103 mAh g^−1^ at −20, −10, 0, and 10°C, respectively. These results further confirm the strong temperature dependence of the cell performance: the capacity is suppressed at deep subzero temperatures but increases markedly as the temperature rises. Long‐term cycling at −35°C demonstrates the durability of the hybrid electrolyte under extreme conditions (Figure 4c). At 0.5 C and −35°C, the LMO/LTP full cell retains 90% of its initial capacity after 700 cycles, with an average Coulombic efficiency of 99.86%. Among the representative studies summarized in Table S3, this corresponds to the longest reported cycling duration at temperatures of −30°C or below. The nearly 100% Coulombic efficiency at low temperature is consistent with the formation of a stable ionic‐liquid‐rich interphase at the anode, which helps suppress parasitic reactions and enhances the reversibility of Li^+^ insertion and extraction in the hybrid electrolyte. Finally, an LED demonstration at −35°C (Figure 4e) shows that the battery can successfully power a 3 V LED, further confirming the practical applicability of the ALIBs based on the [EMIM][DEP]_0.1_‐H_2_O_0.9_ electrolyte in harsh low‐temperature environments.

**FIGURE 5:**
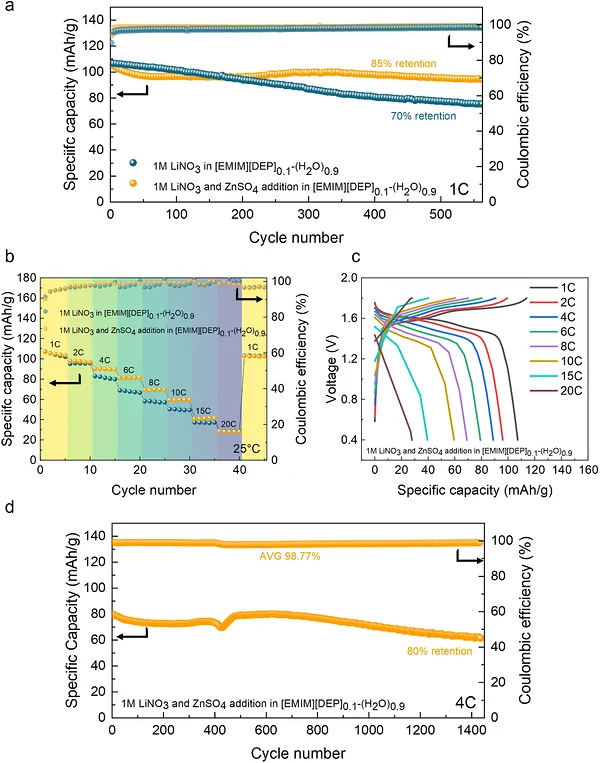
The electrochemical behavior of the LTP/LMO full cell in the hybrid electrolyte at room temperature. (a) Cycling stability of the batteries using 1 M LiNO_3_ in [EMIM][DEP]_0.1_‐(H_2_O)_0.9_ and 1 M LiNO_3_ 0.05 M ZnSO_4_ in [EMIM][DEP]_0.1_‐(H_2_O)_0.9_ at 1C. (b) Rate performance of the batteries using 1 M LiNO_3_ in [EMIM][DEP]_0.1_‐(H_2_O)_0.9_ and 1 M LiNO_3_ 0.05 M ZnSO_4_ in [EMIM][DEP]_0.1_‐(H_2_O)_0.9._ (c) Galvanostatic charge‐discharge profiles at different currents. Cycling tests of the batteries using 1 M LiNO_3_ and 0.05 M ZnSO_4_ in [EMIM][DEP]_0.1_‐(H_2_O)_0.9_ at (d) 4C.

After demonstrating that the [EMIM][DEP]_0.1_‐(H_2_O)_0.9_ electrolyte enables stable low‐temperature operation, we next evaluated the room‐temperature cycling stability and rate capability of LMO/LTP full cells. Figure 5a, Figures S20 and S21 summarize the cycling performance and Coulombic efficiency (CE) of the LMO/LTP full cells at room temperature. The battery using 1 M LiNO_3_ in [EMIM][DEP]_0.1_‐(H_2_O)_0.9_ exhibits a capacity retention of 69% after 800 cycles at 1 C, indicating reasonable but limited long‐term stability. To enhance the cyclic lifetime, ZnSO_4_ was introduced into the electrolyte. With 1 M LiNO_3_ + 0.05 M ZnSO_4_ in [EMIM][DEP]_0.1_‐(H_2_O)_0.9_, the capacity retention is significantly improved to 85% after 550 cycles, showing that the presence of Zn^2^^+^ plays an important role in stabilizing the electrode‐electrolyte interface and suppressing side reactions during prolonged cycling [30]. Importantly, the introduction of 0.05 M ZnSO_4_ has little influence on the low‐temperature capacity, as the discharge capacities from −35°C to 10°C are nearly identical with and without Zn^2^^+^ (Figure S22). During charging, a small amount of Zn^2+^ can be reduced to Zn metal, which exhibits a high overpotential for H_2_ evolution and thus helps to suppress the hydrogen evolution reaction (HER). The reversible redox response of the Zn^2+^‐containing full cell at different scan rates was first confirmed by CV. (Figure S19) The XRD further shows that, after 1400 cycles in the electrolyte containing 0.05 M ZnSO_4_, the characteristic diffraction peaks of LMO remain at essentially the same positions and no Zn‐related reflection is detected on the LMO cathode, whereas weak reflections coinciding with metallic Zn are observed on the LTP electrode (Figure S24a,b). When the ZnSO_4_ concentration is increased to 0.1 M, Zn‐related reflections become detectable on LMO, and the reversible capacity decreases from approximately 107 to approximately 60 mAh g^−1^ (Figure S24c,d). These results support the selection of 0.05 M ZnSO_4_ as an interfacial additive concentration that improves cycling stability without causing XRD‐detectable structural deterioration of LMO. For comparison, the cell with 1 M LiNO_3_ in water shows only 28% capacity retention after 350 cycles (Figure S23), highlighting the combined benefits of the ionic liquid and Zn^2+^ additive for room‐temperature cycling stability. The rate performance of the Zn^2+^‐containing electrolyte was further evaluated at current densities ranging from 1 C to 20 C (Figure 4c,d). The cell delivers average discharge capacities of approximately 107, 95, 89, 79, 69, 59, 39, and 28 mAh g^−1^ at 1 C, 2 C, 3 C, 5 C, 8 C, 10 C, 15 C, and 20 C, respectively. When the current density is returned to 1 C, the capacity is almost fully recovered, indicating excellent rate reversibility. Long‐term high‐rate cycling tests confirm the robustness of this system: after 1400 cycles at 4 C, a high capacity retention of 80% is still observed, with an average CE of 98.77% (Figure 4d). After 1400 cycles, the characteristic reflections of both spinel LMO and LTP remain essentially the same 2θ positions as those of the pristine electrodes, without obvious peak disappearance or substantial peak shift (Figure S24a,b). These results confirm that the bulk crystal structures of both electrodes are preserved during prolonged cycling in the optimized electrolyte. These results demonstrate that the hybrid IL/aqueous electrolyte with Zn^2^^+^ not only provides outstanding cycling stability at room temperature but also supports excellent high‐rate performance. The high and stable Coulombic efficiency is consistent with the formation of an ionic‐liquid‐rich interphase at the anode, which effectively suppresses parasitic reactions. Self‐discharge was evaluated by fully charging matched cells and holding them under open‐circuit conditions for either 20 or 168 h before discharge. After 20 h, the cell using 1 M LiNO_3_ in H_2_O retained 69.6% of its initial discharge capacity. In comparison, the cell using 1 M LiNO_3_ 0.05 M ZnSO_4_ in [EMIM][DEP]_0.1_‐(H_2_O)_0.9_ retained 91.6% (Figure S26a–d). The hybrid electrolyte therefore reduced the capacity loss from 30.4% to 8.4%, corresponding to a 72.4% reduction in self‐discharge‐related capacity loss. After 168 h, the cell using 1 M LiNO_3_ in H_2_O yielded no recoverable discharge capacity within the applied voltage window, corresponding to 0% capacity retention. By contrast, the cell using the ZnSO_4_‐containing hybrid electrolyte retained 44.8% of its initial discharge capacity (Figure S26e–h). These results demonstrate that the hybrid electrolyte substantially suppresses both short‐ and long‐duration self‐discharge. Besides ZnSO_4_ addition, the room‐temperature performance can also be tuned by the [EMIM][DEP] content. With 1 M LiNO_3_ in [EMIM][DEP]_0.2_‐(H_2_O)_0.8_, the cell retained 75% of its initial capacity after 890 cycles at 1 C (Figure S25), indicating that increasing the ionic‐liquid content strengthens the ionic‐liquid‐rich, water‐restricted interfacial region and suppresses water‐induced parasitic reactions. This benefit is temperature dependent: at −35°C and 0.2 C, the 20 mol% formulation delivers approximately 40 mAh g^−1^, whereas the optimized 10 mol% formulation delivers approximately 90 mAh g^−1^ (Figure S16), because the higher ionic‐liquid content increases the activation energy for ion transport from 0.25 to 0.31 eV. At −20°C, the two formulations show comparable performance. The 10 and 20 mol% formulations therefore provide complementary choices for deep‐subzero and moderate/room‐temperature operation, respectively.

## 3 Conclusions

In this work, we developed a dilute [EMIM][DEP]–water hybrid electrolyte containing 10 mol% ionic liquid and 1 M LiNO_3_ for low‐temperature aqueous lithium‐ion batteries. DEP^−^ reorganizes the water hydrogen‐bond network and suppresses freezing, while EMIM^+^/DEP^−^ enrichment near the LTP anode restricts water access and mitigates hydrogen evolution. This combined bulk and interfacial regulation broadens the apparent electrochemical stability window and enables cells to retain 90% capacity after 700 cycles at −35°C. The addition of ZnSO_4_ further improves room‐temperature cycling durability. Conventional water‐in‐salt electrolytes broaden the stability window through highly concentrated salts, but their high viscosity, cost, and risk of low‐temperature precipitation can limit practical operation [13, 14, 15]. Organic‐cosolvent electrolytes depress the freezing point and regulate Li^+^ solvation, but often require large organic fractions that may increase viscosity and partially compromise their predominantly aqueous character [7]. In comparison, the present electrolyte achieves effective antifreezing and interfacial protection using moderate salt concentration and low ionic‐liquid content. These findings establish ionic liquid/water hybridization as a compositionally efficient strategy for aqueous batteries operating across a broad temperature range.

## 4 Experimental Section

### 4.1 Materials

1‐ethyl‐3‐methylimidazolium diethyl phosphate ([EMIM][DEP]) and 1‐Ethyl‐3‐methylimidazolium bis(trifluoromethylsulfonyl)imide ([EMIM][TFSI]) were purchased from Shanghai Cheng Jie Chemical Co., Ltd. LiNO_3_, ZnSO_4_, and 1‐Ethyl‐3‐methylimidazolium tetrafluoroborate ([EMIM][BF_4_]) were obtained from Aladdin. LiMn_2_O_4_ (LMO), polyvinylidene difluoride (PVDF), and acetylene black were supplied by Guangdong Canrd New Energy Technology Co., Ltd. Carbon‐coated LiTi_2_(PO_4_)_3_ (C‐LTP, 3 wt.% carbon) was purchased from Guangdong Qianjin Chemical Reagent Co., Ltd (China).

### 4.2 Preparation of Water/[EMIM][DEP] Electrolyte

The hybrid water/[EMIM][DEP] electrolytes were prepared by mixing [EMIM][DEP] and water at a molar ratio of [EMIM][DEP]: H_2_O = 1:9, 2:8, 3:7, 4:6, 5:5, 6:4, 8:2 and 9:1, respectively. Then, different concentrations LiNO_3_ and ZnSO_4_ were dissolved in the mixture through sufficiently stirring to form the final electrolytes.

### 4.3 Battery Assembly

70 wt.% active materials (LTP and LMO) with 10 wt.% Polyvinylidene fluoride (PVDF), and 20 wt.% carbon black in N‐Methyl‐2‐pyrrolidone (NMP) solution were mixed to form the slurries of cathode and anode. The slurries were evenly coated on circular stainless‐steel sheets with a radius of 0.7 cm followed by baking in a vacuum oven at 100°C for 12 h to get the final electrodes. The final mass loadings of the cathode and anode active materials are 4 and 8 mg/cm^2^, respectively. The specific capacity of the battery was calculated based on the cathode active material. The primary full cell tests used LMO and LTP active‐material loadings of 4 and 8 mg cm^−2^, respectively. Additional mass‐balance control cells were prepared with LMO/LTP loadings of 4/5 mg cm^−2^ and tested at 25°C (1 C) and −35°C (0.2 C) (Figure S18). Unless otherwise stated, the specific capacity is reported based on the active mass of the LMO cathode. On the basis of the combined active masses of both electrodes, the approximate room‐temperature specific capacities are 35.7 mAh g^−1^ for the 4/8 configuration and 47.6 mAh g^−1^ for the 4/5 configuration.

Finally, 2025‐coin cell batteries were assembled using a hybrid aqueous/[EMIM][DEP] electrolyte, LMO cathode, C‐LTP anode, and glass fiber separator.

### 4.4 Molecular Dynamics Simulation

The molecular dynamics system comprises an electrolyte (LiNO_3_, ZnSO_4_, [EMIM][DEP], and water) confined between two electrode slabs (anode: LiTi_2_(PO_4_)_3_; cathode: LiMn_2_O_4_). Different electrical potentials were applied onto the two electrode slabs (anode: 2.5 V; cathode: 4.1 V), and the system pressure was maintained at 1 atm. The length of the simulation box (x) was 10 nm, and the width (y) and height (z) were both 5 nm (Figure S27).

Equilibrium MD simulations were conducted using the LAMMPS package [31]. The electrolyte was simulated in the NVT ensemble at temperatures of 300 K and 238.15 K. The two electrode slabs were simulated in the NVE ensemble without considering lattice vibrations. The electrode potentials were controlled using the constant potential method (CPM) [32, 33]. The OPLS‐AA force field for ionic liquids was used to describe the molecular interactions of [EMIM][DEP] [34]. The SPC/E model was employed to simulate water molecules [35], while the potential parameters of LiNO_3_ and ZnSO_4_ were adopted from the literature [36, 37, 38]. The water molecules, NO_3_^−^ and SO_4_^2−^ were considered rigid. The timestep was set to be 0.5 fs, and the simulation was run for 4 ns, and the last 2 ns was used for data collection.

### 4.5 Characterizations

The Fourier‐transform infrared spectroscopy (FTIR) results of the hybrid aqueous/[EMIM][DEP] were obtained using a Bruker Vertex 70 Hyperion 1000 spectrometer (ATR) at the wavenumber range of 4000 to 400 cm^−1^. Differential scanning calorimetry was conducted with a DSC TA Q 1000. The samples were scanned from 0°C to −100°C at a cooling rate of 5°C/min in N_2_ atmosphere. ^1^H NMR analysis was carried out on a Varian Mercury 300 MHz High Resolution NMR spectrometer. Low‐temperature freezing tests were conducted in a control chamber (LJPTH‐100E) from LIK Group Co., Limited.

### 4.6 Electrochemical Measurements

The electrochemical window of the electrolytes was measured through linear sweep voltammetry (LSV) test at a scan rate of 1 mV/s. Here, a three‐electrode system was used with stainless‐steel mesh as the working and counter electrodes, and Ag/AgCl (in 3 M KCl aqueous solution) as the reference electrode. Then, the electrochemical window was converted to Li/Li^+^ reference for convenience. The long‐cycle performance of the batteries was tested on a battery test system (XINWEI, CT2001A) in a voltage range of 0.4–1.8 V. The low‐temperature performance of the batteries was tested on the LAND CT2001A battery test system with the battery exposed to different temperatures at the voltage range of 0.4–1.9 V. Low‐temperature electrochemical performance tests were in the polycarbonate open bath system. The battery temperature was controlled by the control chamber. The electrochemical impedance spectroscopy (EIS) of the full batteries after different cycles and under different temperatures was performed on a Metrohm Autolab PGSTAT302N electrochemical station. Self‐discharge was evaluated by fully charging matched cells under identical conditions, holding them at open circuit for 20 h while recording the voltage‐time response, and subsequently discharging them under the same conditions. The retained capacity was calculated as the discharge capacity after the open‐circuit rest divided by the discharge capacity obtained without the rest period.

### 4.7 Statistical Analysis

The date for the battery, including galvanostatic charge–discharge (GCD) and electrochemical impedance spectroscopy (EIS), were collected through the XINWEI battery test system and Metrohm Autolab PGSTAT302N electrochemical station. The data were processed from the mean of three sets of experimental data. For the temperatures of the battery was recorded by the handheld thermometer. For software used for statistical analysis, commercial software PowerPoint and Origin were used to process the data.

## Author Contributions

**Jiwei He**: conceptualization, methodology, validation, investigation, funding acquisition, visualization, writing – review and editing, writing – original draft, project administration, formal analysis, software, data curation, resources. **Le Zhou**: methodology, software, Writing – original draft, validation, visualization, data curation, resources. **Mengmeng Cong**: investigation, methodology, validation, formal analysis, visualization, resources. **Keqiao Li**: investigation, methodology, validation, formal analysis, resources. **Donggyu Lee**: investigation, visualization, validation, resources. **Jiacheng Ji**: investigation, validation, visualization, resources. **Jinyu Deng**: investigation, validation, visualization, resources. **Zhigang Li**: writing – review and editing, supervision, resources, project administration. **Baoling Huang**: funding acquisition, writing – review and editing, conceptualization, investigation, project administration, supervision, methodology.

## Conflicts of Interest

The authors declare no conflicts of interest.

## Supporting information




**Supporting File**: advs77610‐sup‐0001‐SuppMat.docx

## Data Availability

The data that support the findings of this study are available from the corresponding author upon reasonable request.
