# Supplementary material for: Water/Ionic Liquid Hybrid Electrolyte for High Cycling Stability and Low‐Temperature Adaptability in Aqueous Lithium‐Ion Battery
Source: Adv Sci (Weinh). 2026 Sep 10:e77610. Online ahead of print. doi: 10.1002/advs.77610 (PMC13562784; doi:10.1002/advs.77610)
Supplement: Supplementary file 1 [file ADVS-9999-e77610-s001.docx]

**Fig. S1** Photographs of different ionic liquid/water mixtures after 1 h at different temperatures. (From left to right: 1M LiNO_3_ in water, [EMIM][BF_4_]_0.1_-(H_2_O)_0.9_, [EMIM][TFSI]_0.1_-(H_2_O)_0.9_, [EMIM][DEP]_0.1_-(H_2_O)_0.9_ and 1M LiNO_3_ in [EMIM][DEP]_0.1_-(H_2_O)_0.9_.)

**Fig. S2** Photograph of the electrolytes of 1 M LiNO_3_, Li_2_SO_4_ and LiTFSI in [EMIM][DEP]_0.1_-(H_2_O)_0.9_.

**Fig. S3** Evaluation of alternative lithium salts in the [EMIM][DEP]-water hybrid electrolyte. (a) Photograph of 1 M LiTFSI in [EMIM][DEP]_0.1_-H_2_O_0.9_ after storage at -35 °C. (b) DSC curve of 1 M LiCl in [EMIM][DEP]_0.1_-H_2_O_0.9_. (c) Photographs of 1 M LiCl in [EMIM][DEP]_0.1_-H_2_O_0.9_ after storage at the indicated temperatures.

**Fig. S4** DSC curves of [EMIM][DEP]_0.2_–H_2_O_0.8_ and 1M LiNO_3_ in [EMIM][DEP]_0.2_-H_2_O_0.8._

**Fig. S5** Temperature-dependent ionic conductivity of [EMIM][DEP]-H_2_O mixtures with different molar ratios.

**Fig. S6** FTIR spectra of [EMIM][DEP]-H_2_O mixtures with varying [EMIM][DEP] molar fractions between 4000-400 cm^-1^

**Fig. S7** ^1^H NMR spectra for [EMIM][DEP]_0.3_-(H_2_O)_0.7_

**Fig. S8** ^1^H NMR spectra for [EMIM][DEP]_0.2_-(H_2_O)_0.8_

**Fig. S9** ^1^H NMR spectra for [EMIM][DEP]_0.1_-(H_2_O)_0.9_

**Fig. S10** Study of the hydrogen bond in the hybrid electrolyte. MD simulation snapshots of H_2_O at (a) 300K and (b) 238K. The RDFs of the oxygen atom in water molecules at (c) 300K and 238K.

**Fig. S11** The LSV of 1M LiNO_3_ in pure water.

**Fig. S12** MD simulation snapshots of [EMIM][DEP]_0.1_-(H_2_O)_0.9_ at 238K.

**Fig. S13** CV profiles collected in the hybrid electrolyte at for LMO and LTP.

**Fig. S14** Photograph of the low temperature battery test is obtained on a high-low temperature test chamber.

**Fig. S15** Experimental and fitted temperature-dependent EIS spectra of the separator-only cells (a,b) and LMO/LTP full cells (c,d) using 1 M LiNO_3_ in [EMIM][DEP]_0.1_-H_2_O_0.9_ (a,c) and 1 M LiNO_3_ + 0.05 M ZnSO_4_ in [EMIM][DEP]_0.1_-H_2_O_0.9_ (b,d). The corresponding equivalent circuit models are shown in the insets.

**Fig. S16** Galvanostatic charge-discharge profiles for 1 M LiNO_3_ [EMIM][DEP]_0.2_-H_2_O_0.8_ (a) at 238K at 0.2C and (b) at 253K at 0.2C.

**Fig. S17** Galvanostatic charge-discharge profiles at different temperature at 0.5C for 1M LiNO_3_ in pure water

**Fig. S18** Charge-discharge profiles of LMO/LTP full cells with LMO/LTP active-material loadings of 4/5 mg cm^-2^ measured at (a) 25 °C and 1 C and (b) -35 °C and 0.2 C. The specific capacities are based on the active mass of the LMO cathode.

**Fig. S19** CV profiles of the batteries using 1 M LiNO_3_ 0.05M ZnSO_4_ in [EMIM][DEP]_0.1_-(H_2_O)_0.9_ electrolyte at different scan rates at room temperature.

**Fig. S20** The corresponding coulombic efficiencies of LMO/LTP full cell for 1M LiNO3 in [EMIM][DEP]_0.1_-H_2_O_0.9_

**Fig. S21** The corresponding coulombic efficiencies of LMO/LTP full cell for 1M LiNO3 0.05M ZnSO_4_ in [EMIM][DEP]_0.1_-(H_2_O)_0.9._

**Fig. S22** The electrochemical performance of full batteries with the hybrid electrolyte at 0.2C under different temperatures for Zn^+^ addition [EMIM][DEP]_0.1_-(H_2_O)_0.9_ and [EMIM][DEP]_0.1_-(H_2_O)_0.9_

**Fig. S23** Cycling performance of the full cell of LMO/LTP full cell for 1M LiNO_3_ in H_2_O_._

**Fig. S24** Ex situ XRD patterns and concentration-dependent influence of ZnSO_4_: (a) LMO and (b) LTP electrodes before cycling and after 1,400 cycles in 1 M LiNO_3_ + 0.05 M ZnSO_4_ in [EMIM][DEP]_0.1_-(H_2_O)_0.9_; (c) LMO before and after cycling in 1 M LiNO_3_ + 0.1 M ZnSO_4_ in [EMIM][DEP]_0.1_-(H_2_O)_0.9_; and (d) comparison of the charge-discharge profiles of LMO/LTP full cells using 0.05 and 0.1 M ZnSO_4_. Dashed lines indicate the reference diffraction positions of metallic Zn.

**Fig. S25** Room-temperature cycling stability of the LMO||LTP full cell using 1 M LiNO_3_ in [EMIM][DEP]_0.2_-(H_2_O)_0.8_ at 1 C and 298 K.

**Figure S26.** Self-discharge behavior of LMO/LTP full cells using different electrolytes. (a,b) Voltage–time profiles during a 20 h open-circuit rest and (c,d) the corresponding charge–discharge profiles before and after the rest. (e,f) Voltage–time profiles during a 168 h open-circuit rest and (g,h) the corresponding charge–discharge profiles before and after the rest. Panels (a,c,e,g) correspond to 1 M LiNO_3_ in H_2_O, whereas panels (b,d,f,h) correspond to 1 M LiNO_3_ in [EMIM][DEP]_0.1_–(H_2_O)_0.9_. The red dashed lines indicate the discharge capacities recovered after the specified open-circuit resting periods.

**Fig. S27** Molecular dynamics simulation system.

**Table S1** Summary of battery performance with different organic additives

| **Organic additives** | **Molar ratio(%)** | **Salt** | **Conductivity (mS/cm^-1^)** | **Hazards** | **Reference** |
| --- | --- | --- | --- | --- | --- |
| [EMIM][DEP] | 10 | 1M LiNO_3_ | 1.20 at -40°C |  | This work |
| Dimethyl ether | 15 | 1M Zn(OTf)_2_ | 1.06 at -40°C |  | [1] |
| Formamide | 31 | 1M Zn(OAc)_2_ | 1.15 at -40°C |  | [2] |
| 1,3-dioxolane | 32 | 10M LiTFSI | 0.9 at -40°C |  | [3] |
| Acetonitrile | 47 | 1M Zn(CF_3_SO_3_)_2_ | 28.6 at -40°C |  | [4] |
| Dimethyl sulfoxide | 30 | 2M NaClO_4_ | 0.11 at -50°C |  | [5] |
| Ethylene glycol | 16 | 1M Li_2_SO_4_ | 4.2 at -20°C |  | [6] |

**Table S2** Comparison of the low-temperature cycling performance of representative aqueous lithium-based batteries.

| Electrolyte composition | Temperature (°C) | Cycling rate | Cycle number | Capacity retention | Reference |
| --- | --- | --- | --- | --- | --- |
| 1 M LiNO_3_ in EMIMDEP_0.1_-H_2_O_0.9_ | -35 | 0.5 C | 700 | 90% | This work |
| 9 M LiTFSI in H_2_O/DMF with 5 wt% 3-sulfolene | -30 | 0.3 C | 50 | 98.8% | [7] |
| Sulfolane-H_2_O-LiClO4 | -20 | 200 mA g^-1^ | 200 | 87% | [8] |
| 10 M LiTFSI in H2O/DOL, 1:1 | -20 | 0.5 C | 100 | 94% | [3] |
| 1.1 M lithium methanesulfonate-TMP-H_2_O | -20 | 0.2 C | 100 | Approximately 100% | [9] |
| 1 M Li_2_SO_4_ with 20 wt% ethylene glycol | -20 | 1 C | 100 | 85.8% | [6] |
| Concentrated LiFSI in H_2_O/DMSO | -10 | N.R. | 50 | 72.2% | [10] |
| 15.3 M LiTFSI in H_2_O/AN, 1:1 | 0 | 1 C | 120 | 95% | [11] |

**Table S3** Fitted resistance components obtained from the temperature-dependent EIS spectra of the full cells.

| Electrolyte | Temperature (°C) | R_b_ (Ω) | Rct,cell (Ω) |
| --- | --- | --- | --- |
| 1 M LiNO_3_ in [EMIM][DEP]_0.1_-H₂O_0.9_ | 25 | 2.74 | 6.29 |
|  | 10 | 4.42 | 7.11 |
|  | 0 | 6.57 | 12.21 |
|  | -10 | 10.35 | 14.90 |
|  | -20 | 20.04 | 27.02 |
|  | -35 | 42.19 | 39.44 |
| 1 M LiNO_3_ + 0.05 M ZnSO_4_ in [EMIM][DEP]_0.1_-H_2_O_0.9_ | 25 | 2.84 | 8.91 |
|  | 10 | 4.02 | 12.40 |
|  | 0 | 7.43 | 15.68 |
|  | -10 | 9.45 | 24.02 |
|  | -20 | 18.98 | 34.69 |
|  | -35 | 34.39 | 45.35 |

**Reference**

[1]	Y. Dong, N. Zhang, Z. Wang, J. Li, Y. Ni, H. Hu, F. Cheng, *Journal of Energy Chemistry* **2023**, *83*, 324.

[2]	C. You, R. Wu, X. Yuan, L. Liu, J. Ye, L. Fu, P. Han, Y. Wu, *Energy & Environmental Science* **2023**, *16*, 5096.

[3]	Z. Ma, J. Chen, J. Vatamanu, O. Borodin, D. Bedrov, X. Zhou, W. Zhang, W. Li, K. Xu, L. Xing, *Energy Storage Materials* **2022**, *45*, 903.

[4]	J. Wang, Q. Zhu, F. Li, J. Chen, H. Yuan, Y. Li, P. Hu, M. S. Kurbanov, H. Wang, *Chemical Engineering Journal* **2022**, *433*, 134589.

[5]	Q. Nian, J. Wang, S. Liu, T. Sun, S. Zheng, Y. Zhang, Z. Tao, J. Chen, *Angewandte Chemie International Edition* **2019**, *58*, 16994.

[6]	A. Tron, S. Jeong, Y. D. Park, J. Mun, *ACS Sustainable Chemistry & Engineering* **2019**, *7*, 14531.

[7]	Z. Ma, Z. Xie, J. Liu, J. Vatamanu, L. Xing, W. Li, *Energy Storage Materials* **2024**, *66*, 103203.

[8]	J. Liu, C. Yang, X. Chi, B. Wen, W. Wang, Y. Liu, *Advanced Functional Materials* **2022**, *32*, 2106811.

[9]	J. Zhi, Y. Luo, C. Liao, Z. Liu, M. Han, K. Yue, L. Zhang, G. Li, P. Chen, *Research* **2026**, *9*, 1170.

[10]	I. C. Pellini, N. Pianta, D. Callegari, G. Conte, R. Lorenzi, C. Simari, M. De Bonis, S. Leonardi, Z. Jusys, J. C. Espinosa-Angeles, *Electrochimica Acta* **2025**, 146903.

[11]	J. Chen, J. Vatamanu, L. Xing, O. Borodin, H. Chen, X. Guan, X. Liu, K. Xu, W. Li, *Advanced Energy Materials* **2020**, *10*, 1902654.
